# Bicarbazole-Benzophenone Based Twisted Donor-Acceptor Derivatives as Potential Blue TADF Emitters for OLEDs

**DOI:** 10.3390/molecules29071672

**Published:** 2024-04-08

**Authors:** Iram Siddiqui, Prakalp Gautam, Dovydas Blazevicius, Jayachandran Jayakumar, Sushanta Lenka, Daiva Tavgeniene, Ernestas Zaleckas, Saulius Grigalevicius, Jwo-Huei Jou

**Affiliations:** 1 Department of Materials Science and Engineering, National Tsing Hua University, Hsinchu 30044, Taiwanjayakumar@mx.nthu.edu.tw (J.J.); sushantalenka1@gmail.com (S.L.); 2 Department of Polymer Chemistry and Technology, Kaunas University of Technology, Radvilenu Plentas 19, LT-50254 Kaunas, Lithuaniadaiva.tavgeniene@ktu.lt (D.T.); 3 Department of Agricultural Engineering and Safety, Agriculture Academy, Vytautas Magnus University, Studentu Str. 11, Akademija, LT-53361 Kaunas, Lithuania

**Keywords:** organic light-emitting diode (OLED), blue TADF emitters, Donor-Acceptor (D-A) materials, benzophenone-based derivatives

## Abstract

Over the past few decades, organic light-emitting diodes (OLEDs) find applications in smartphones, televisions, and the automotive sector. However, this technology is still not perfect, and its application for lighting purposes has been slow. For further development of the OLEDs, we designed twisted donor-acceptor-type electroactive bipolar derivatives using benzophenone and bicarbazole as building blocks. Derivatives were synthesized through the reaction of 4-fluorobenzophenone with various mono-alkylated 3,3′-bicarbazoles. We have provided a comprehensive structural characterization of these compounds. The new materials are amorphous and exhibit suitable glass transition temperatures ranging from 57 to 102 °C. They also demonstrate high thermal stability, with decomposition temperatures reaching 400 °C. The developed compounds exhibit elevated photoluminescence quantum yields (PLQY) of up to 75.5% and favourable HOMO-LUMO levels, along with suitable triplet-singlet state energy values. Due to their good solubility and suitable film-forming properties, all the compounds were evaluated as blue TADF emitters dispersed in commercial 4,4′-bis(N-carbazolyl)-1,10-biphenyl (CBP) host material and used for the formation of emissive layer of organic light-emitting diodes (OLEDs) in concentration-dependent experiments. Out of these experiments, the OLED with 15 wt% of the emitting derivative 4-(9′-{2-ethylhexyl}-[3,3′]-bicarbazol-9-yl)benzophenone exhibited superior performance. It attained a maximum brightness of 3581 cd/m^2^, a current efficacy of 5.7 cd/A, a power efficacy of 4.1 lm/W, and an external quantum efficacy of 2.7%.

## 1. Introduction

In recent decades, there has been significant and swift advancement in organic light-emitting diode (OLED) technology, transforming it into a multi-billion-dollar market [1]. Its applications have expanded across various domains, encompassing high-contrast flat-panel displays, smartwatches, smartphones, and big-screen television sets. In addition, solid-state lighting is attracting growing interest in both industrial and scientific domains [2,3,4,5,6,7]. OLED devices offer superior features such as high colour purity, reduced weight, lower power consumption, faster response, and flexibility, surpassing capabilities offered by existing technologies [8,9,10,11]. 

Until now, the prevailing commercial OLED devices were based on phosphorescent materials containing noble metals like platinum and iridium [12]. The incorporation of atoms of noble metals into the structures of phosphorescent materials presents a notable obstacle not only to the future manufacturing expenses of devices but also prompts environmental concerns [13,14]. Moreover, there is a tendency to increase the nonradiative transition rate of phosphorescent metal complex d-orbitals when the emission peaks are shifting to the blue region of emission, posing challenges in achieving both high efficiency and stability in blue phosphorescent OLEDs [15,16,17,18,19,20]. To address these challenges, there is a renewed focus on the development of small-molecule fluorescent materials, primarily due to their high colour purity and cost-effectiveness [21]. In recent years, there has also been considerable attention focused on thermally activated delayed fluorescence (TADF) materials. This is due to absence of metal atoms in their structures and their capability to employ reverse intersystem crossing (RISC), resulting in the up-conversion of triplet excitons to emissive singlet excitons, leading to significantly enhanced external quantum efficiencies (EQEs) [22,23,24,25,26,27,28]. However, numerous TADF OLEDs face challenges including triplet-triplet and singlet-triplet annihilation, as well as concentration quenching. These issues can be attributed to prolonged exciton lifetimes, leading to a notable decrease in efficiency as luminance increases [29,30,31].

A crucial requirement for TADF OLED emitters to function efficiently is achieving the smallest possible singlet-triplet energy splitting (ΔE_ST_). This can be accomplished through molecular design strategies aimed at maximizing the separation between the highest occupied molecular orbital (HOMO) and the lowest unoccupied molecular orbital (LUMO). One effective approach is the incorporation of highly twisted donor and acceptor structure in the derivatives [30,32,33]. Benzophenone and its derivatives having strong electron-withdrawing capabilities, effective intersystem crossing due to robust spin-orbit coupling and twisted configuration offer the potential for developing efficient TADF emitters with shorter exciton lifetimes through smart molecular design [34,35,36,37,38,39,40,41,42]. Carbazole derivatives, widely recognized for their electron-rich nature, were extensively employed as electron donors in a wide range of optoelectronic devices, serving as both host materials and emitters in various configurations. This is due to the capacity of 9H-carbazole for facile functionalization across multiple sites, tuneable electronic and optical properties, robust electrochemical and thermal stability, and high photoluminescence quantum yield [43,44,45,46,47,48,49]. Materials exhibiting both favourable film-forming properties and solubility in common organic solvents are extensively explored in the scientific and technological sectors. This interest stems from the fact that low molar-mass organic derivatives, also called molecular glasses, demonstrate capability to form transparent, stable, and homogenous amorphous layers from their solutions [50,51]. Both benzophenone-based as well as carbazole-based derivatives are valued for their ability to create stable amorphous layers characterized by high glass transition temperatures as reported in the literature [52,53]. Solubility in common organic solvents of new materials enables solution-based manufacturing processes, such as blade or spin coating, and inkjet printing, which are simpler, more cost-effective, and more scalable than the usual vacuum evaporation method [54,55,56,57]. 

In this study, we present the synthesis, investigation, and application of new bipolar electroactive compounds with benzophenone and 3,3′-bicarbazole fragments acting as electron acceptors and electron donors, respectively. The donor-acceptor type twisted molecules demonstrated their efficacy as blue TADF emitters in organic LEDs. The selection of alkyl sidechains, including ethyl, butyl, pentyl, hexyl, 2-ethylhexyl, and octyl was aimed at optimizing their film-forming properties, solubility, and solution processability [58]. 

## 2. Results and Discussion

### 2.1. Synthesis

**Scheme 1:**
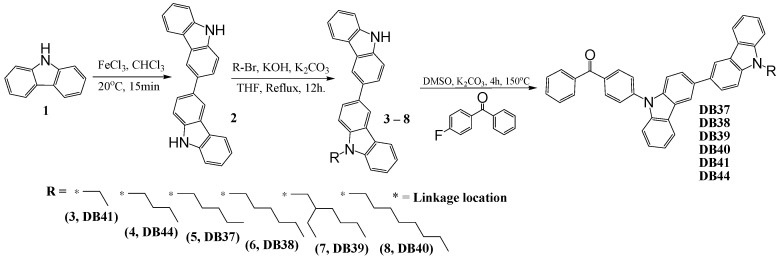
Synthesis of target compounds **DB37**, **DB38**, **DB39**, **DB40**, **DB41** and **DB44**.

Novel electroactive bicarbazole-based derivatives were synthesized via a three-step procedure illustrated in Scheme 1. Initially, 9H-carbazole underwent oxidation with iron (III) chloride to yield 9H,9′H-3,3′-bicarbazole (**2**). Subsequently, various alkyl bromides were utilized for mono-alkylation of the 9H,9′H-3,3′-bicarbazole (**2**) in THF solution, resulting in the production of 9-alkyl-9′H-3,3′-bicarbazoles (**3**–**8**) in the presence of potassium hydroxide and potassium carbonate. Finally, the obtained bicarbazole derivatives (**3**–**8**) underwent nucleophilic substitution reactions with 4-fluorobenzophenone in DMSO in the presence of potassium carbonate, leading to the formation of the target derivatives **DB37**, **DB38**, **DB39**, **DB40**, **DB41**, and **DB44**. The chemical structures of these new electroactive compounds were confirmed using mass spectrometry and NMR spectroscopy, demonstrating excellent alignment with the theoretical structures. The aliphatic chains present in the synthesized target compounds contributed to increased solubility in commonly used organic solvents, consistent with the findings of Inoue et al. regarding the relationship between alkyl chain length and the solubility of organic materials [56]. The solubility of the presented materials in appropriate solvents was enhanced by extending the length of the alkyl chain. While the thermal evaporation method could be suitable for the formation of thin layers for devices using these electroactive compounds, the good solubility of the new materials allows a cost-effective alternative method for forming thin films through spin coating from their solutions.

### 2.2. Thermal and Morphological Properties

**Figure 1:**
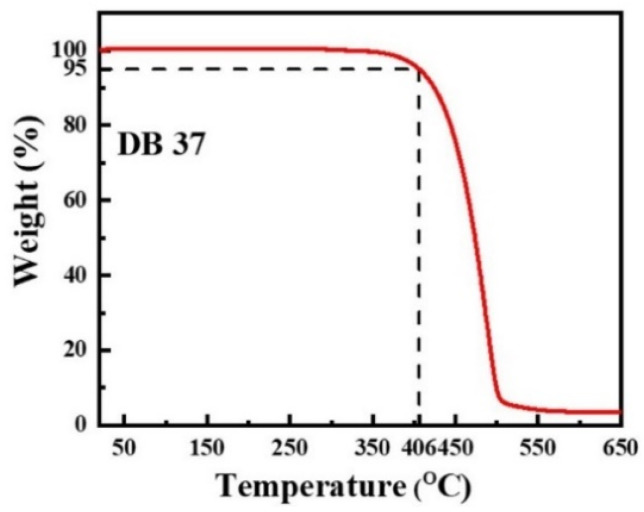
TGA curve (red) of compound **DB37**. Heating rate: 10 °C/min.

The response of the synthesized materials **DB37**–**DB41** and **DB44** to heating was investigated using DSC and TGA methods, heating the samples under an inert nitrogen atmosphere. Following TGA experiments conducted at a heating rate of 10 °C/min. It was observed that the target compounds exhibit remarkable stability under heating. As depicted in Figure 1, the TGA curve of compound **DB37** illustrates a temperature of 5% weight loss (T_d_) at 406 °C. Similarly, derivatives **DB41** and **DB44** demonstrated stability under heating with respective T_d_ values of 374 °C and 389 °C. Materials **DB38**, **DB39**, and **DB40**, which feature longer aliphatic groups, exhibited comparable thermal stability, reaching T_d_ values of 398 °C, 383 °C, and 397 °C, respectively. The TGA curves of all the investigated derivatives are provided in Figure S1 of the Supplementary Material for the publication.

**Figure 2:**
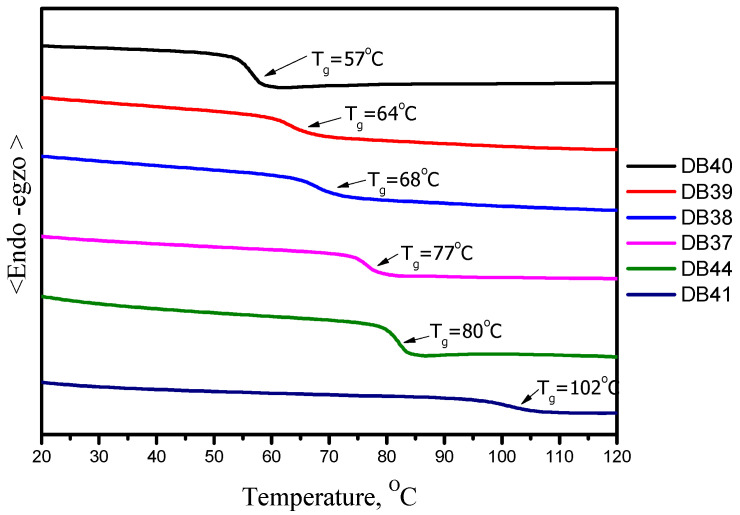
DSC thermograms from the second heating cycle of the target compounds. Heating rate: 10 °C/min.

Figure 2 displays the thermograms of DSC experiments conducted forn all the compounds **DB37**–**DB41** and **DB44**, with sample sizes varying from 2.6 to 4.8 mg. Upon examining the second heating curve, it becomes evident that the glass transition temperatures (T_g_) are influenced by the length of the alkyl sidechains of the compounds. For instance, material **DB41**, containing an ethyl group, exhibited a notably high T_g_ of 102 °C, determined by a slow endothermic dip in the curve of the second heating. Conversely, compounds **DB44** and **DB37**, which were substituted with butyl and pentyl groups, respectively, displayed slightly lower T_g_ values of 80 °C and 77 °C. This trend persists for materials featuring even longer alkyl groups: derivatives **DB38**, **DB39**, and **DB40**, substituted with hexyl, 2-ethylhexyl, and octyl groups respectively, exhibited glass transition temperatures of 68 °C, 64 °C, and 57 °C. This phenomenon could be explained by reduced intermolecular hydrogen bonding as length of the alkyl chain increases [59]. In summary, the findings from the TGA and DSC experiments affirm the suitability of these materials for amorphous electroactive layers of OLED devices. All the thermal characteristics are also presented in Table 1.

### 2.3. Electrochemical and Photo-Physical Properties

**Figure 3:**
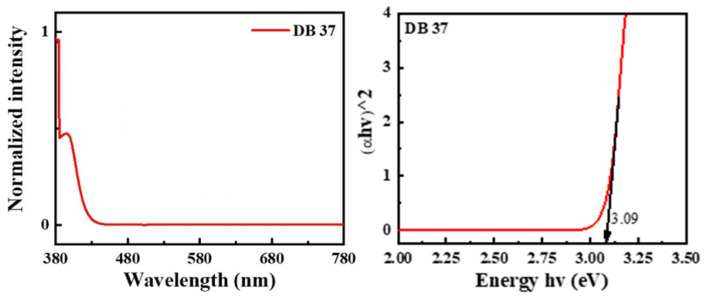
UV-Vis absorbance spectrum (**left**) and tauc plot (**right**) of the compound **DB37**.

The compounds **DB37**, **DB38**, **DB39**, **DB40**, **DB41**, and **DB44** demonstrate elevated photoluminescence quantum yields (PLQY) of 65.5%, 45.3%, 75.5%, 52.5%, 62.5%, and 68.5%, respectively. Summarized values of PLQY can be found in Table 1. Figure 3 illustrates the UV-absorption bands of compound **DB37** as an example. All the UV-absorption bands and Tauc plots for all the compounds are illustrated in Figure S2 of the Supplementary Material of the article. The derivatives were examined in THF solvent under standard conditions using a quartz cuvette. 

Notably, each of the derivatives consistently displayed two absorption peaks around 380 and 410 nm, attributed to the presence of identical chromophores within their structures. Tauc plots for objective compounds were generated by employing the UV absorption wavelength and intensity using equations (α × hν)^1/2^ and hν for the x-axis and y-axis, respectively, where α denotes intensity and hν stands for energy (hν = 1240/wavelength), as it is described in literature [60]. The Tauc plots unveiled bandgaps for the studied derivatives: **DB37**, **DB38**, and **DB41** had a bandgap of 3.09 eV, **DB40** had a bandgap of 3.10 eV, **DB39** had a bandgap of 3.08 eV, and **DB44** had a bandgap of 3.07 eV (see Table 1). The bandgap energy exhibited by the materials was nearly identical, with a maximum difference of 0.03 eV, which aligns closely with the possible measurement discrepancies. Similar bandgap values are acceptable since all the derivatives utilize the same chromophores.

**Figure 4:**
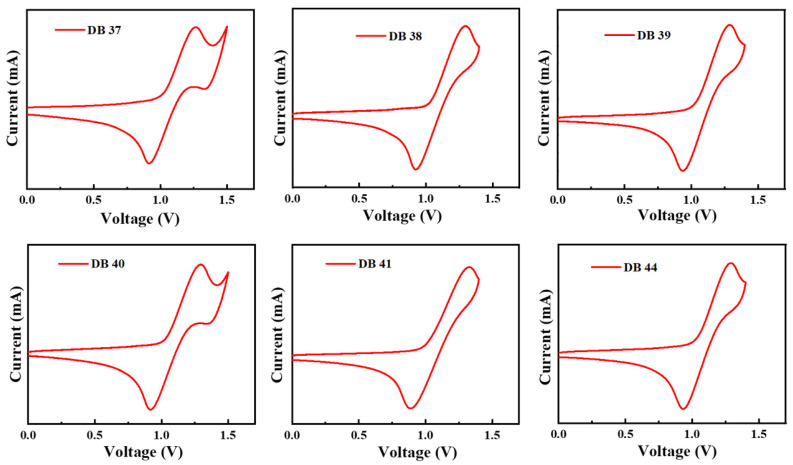
Curves of CV scans of the materials **DB37**, **DB38**, **DB39**, **DB40**, **DB41**, and **DB44**.

Figure 4 illustrates the evaluation of the electrochemical characteristics of **DB37**, **DB38**, **DB39**, **DB40**, **DB41**, and **DB44** through CV measurements. Obtained oxidation onset values were used for calculations of HOMO levels, employing equation E_HOMO_ = −[4.4 + $E_{onset}^{ox}$], while the determination of LUMO levels was accomplished using equation E_LUMO_ = E_HOMO_ + E_g_ following the methodology described in the literature [18,61,62]. The determined HOMO levels for **DB37**, **DB38**, **DB39**, **DB40**, **DB41**, and **DB44** were −5.67, −5.70, −5.68, −5.69, −5.73, and −5.69, respectively. Meanwhile, LUMO levels were, in the same order, −2.58, −2.61, −2.60, −2.59, −2.64, and −2.62. These values, along with E_g_ levels, are outlined in Table 1. HOMO and LUMO levels of the compounds are appropriate for forming blue-emitting layers in tandem with the commercial host material CBP.

**Figure 5:**
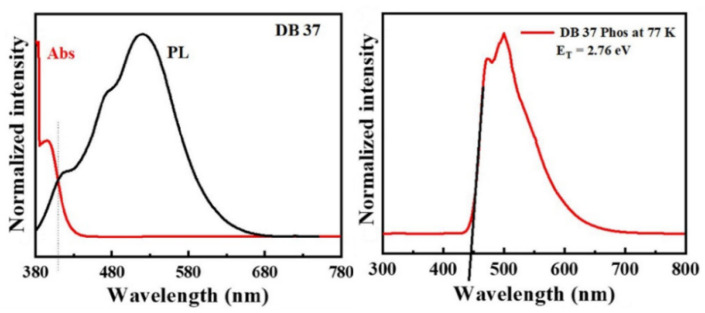
Photoluminescence (**left**) and low-temperature photoluminescence (**right**) spectra of the material **DB37**.

In Figure 5 (left), the PL spectrum of the **DB37** compound is presented, displaying emission wavelength maximum at about 510 nm with cyan blue emission. Singlet state energies of the potential emitters were calculated by utilizing the crossing points of PL and absorbance charts, resulting in values of 3.04 eV for **DB37**, 2.94 eV for **DB38**, 3.10 eV for **DB39**, 3.06 eV for **DB40**, 3.22 eV for **DB41**, and 3.18 eV for **DB44** (see Table 1). 

Additionally, low-temperature photoluminescence (LTPL) spectra were registered to ascertain the triplet energy levels. The spectrum for **DB37** is depicted in Figure 5 (right) as an example. The compounds **DB37**, **DB38**, **DB39**, **DB40**, **DB41**, and **DB44** demonstrate elevated levels of triplet energy at 2.76, 2.89, 2.81, 2.80, 2.80, and 2.82 eV, respectively, suggesting their potential suitability as blue emitters. The LTPL spectra of all the objective derivatives are presented in Figure S3 of Supplementary Material, and the triplet state energy values are listed in Table 1.

**Figure 6:**
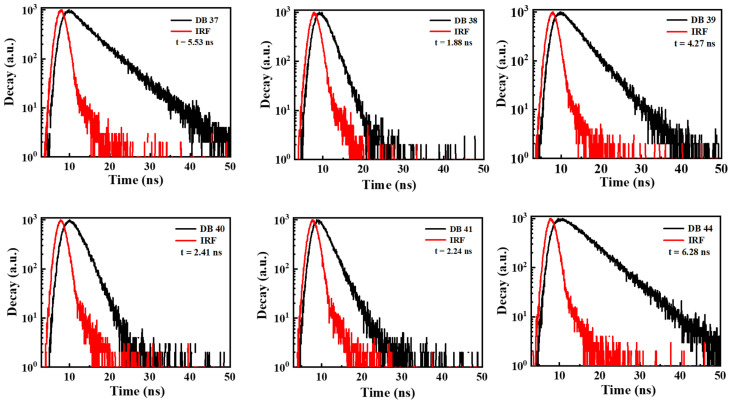
TRPL spectra of the compounds **DB37**, **DB38**, **DB39**, **DB40**, **DB41**, and **DB44**.

Figure 6 displays the results of the time-resolved photoluminescence (TRPL) experiments illustrating the decay times of photoluminescence for the new emitters. The determined values of the time for **DB37**, **DB38**, **DB39**, **DB40**, **DB41**, and **DB44** were 5.53, 1.88, 4.27, 2.41, 2.24, and 6.28 ns, respectively. Typically, the decay lifetime of fluorescent emitters falls within the picosecond range. However, the presented materials exhibit decay on the nanosecond scale, suggesting the potential utilization of triplet excited states as TADF-based emitters [63,64]. The photoluminescence decay times are detailed in Table 1. In the graphs, IRF denotes the instrument response function, which was measured both prior to and following each measurement as a control parameter.

### 2.4. Electroluminescent Properties

**Figure 7:**
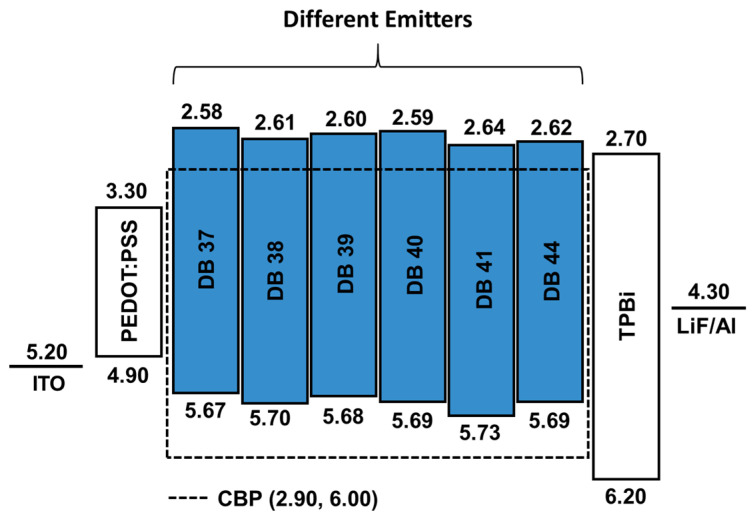
Energy-level diagram of the OLEDs employing the emitters **DB37**, **DB38**, **DB39**, **DB40**, **DB41**, and **DB44** doped in the CBP host.

The OLED device architecture utilized in this study is represented by the energy level diagram depicted in Figure 7. These devices incorporate emitters **DB37**, **DB38**, **DB39**, **DB40**, **DB41**, and **DB44** doped in a CBP host material. The straightforward device structures consisted of a 125 nm ITO anode layer, followed by a 35 nm PEDOT:PSS hole injection layer (HIL), and subsequently a 30 nm emissive layer (EML) comprising a CBP host with dopants **DB37**, **DB38**, **DB39**, **DB40**, **DB41**, or **DB44** (at concentrations of 5%, 10%, 15%, and 100% by weight). As for the electron transporting layer (ETL), 1,3,5-tris(N-phenyl-benzimidazol-2-yl)benzene (TPBi, 32 nm) was employed, while lithium fluoride (LiF, 0.8 nm) served as the electron injecting layer (EIL), and aluminium (Al, 150 nm) was used as the cathode layer.

**Figure 8:**
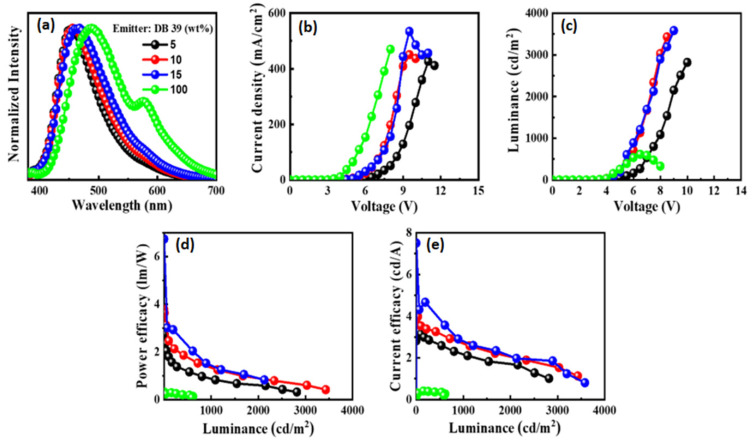
The EL characteristics of the device with pure emitter DB39 or doped within a CBP host material at different concentrations of the DB39: EL spectra (**a**), current density-voltage (**b**), luminance-voltage (**c**), power efficiency-luminance (**d**), and current efficiency-luminance (**e**) characteristics.

All the new objective compounds, owing to their solubility, were suitable for layer preparation through spin-coating and were examined as emitters dispersed in a CBP host for the OLEDs. All new emissive materials underwent concentration-dependent experiments with proportions of 5, 10, 15, or 100 wt% of each guest in the emissive layer. The electroluminescence (EL) properties, like power efficacy (PE), current efficacy (CE), EQE, maximum luminance (L_MAX_), and the International Commission on Illumination (CIE) colour space coordinates of the devices utilizing the newly introduced emitting materials distributed within the CBP host, along with the respective non-doped devices, are outlined in Table 2. Furthermore, Figure 8 visually represents the EL characteristics of devices employing the most efficient emitter, **DB39**. The Figure illustrates the EL spectra of the devices, current density-voltage-luminance, and power efficacy-luminance-current efficacy characteristics. The same characteristics of OLEDs using other emitters **DB37**, **DB38**, **DB40**, **DB41**, and **DB44** are depicted in Figures S4–S8 in the Supplementary Material of this article.

In Figure 8a, the EL spectra of devices incorporating the **DB39** dopant demonstrate peaks within the 460–490 nm range, indicating emission in the blue region. The absence of additional peaks implies effective energy transfer from the host to the guest. Evidently, both undoped and doped OLEDs demonstrate comparable EL emission peaks. Figure 8b–e illustrate the characteristics of current density, luminance, voltage and power efficiency-luminance-current efficiency. The undoped device exhibits a higher current density than the doped devices and correspondingly demonstrates lower efficiency than the doped devices, highlighting the significant influence of the host material. As depicted in Table 2, the OLED based on **DB39** displays the best efficiencies out of all these devices. This enhanced performance can be attributed to the inclusion of the elongated and branched 2-ethylhexyl sidechain in the molecule, potentially improving solubility for the production of wet-processed OLEDs and contributing to the favourable film-forming characteristics of the derivative [65]. Moreover, appropriate HOMO and LUMO levels facilitate effective energy transfer from host to dopant, while the combination of the electron-accepting benzophenone fragment with the bicarbazole donor moiety promotes balanced charge transfer and efficient utilization of excitons [66,67]. Specifically, the device containing 10 wt% of emitter **DB39** demonstrates the highest PE of 4.4 lm/W with a L_MAX_ reaching 3430 cd/m^2^. However, the overall best efficiency is achieved by the device incorporating 15wt% of emitter **DB39** in its emissive layer, attaining PE and CE values of 4.1 lm/W and 5.7 cd/A, respectively, while EQE reached 2.7% with L_MAX_ of 3581 cd/m^2^. The findings of this study indicate the potential utility of benzophenone and bicarbazole fragments in the synthesis of organic semiconductors and also demonstrates how thermal and film-forming properties could be controlled by introducing and modifying alkyl chains within the molecular structure of the new materials.

## 3. Materials and Methods

### 3.1. Instrumentation

The recording of ^1^H and ^13^C nuclear magnetic resonance (NMR) spectra was conducted with the Bruker Avance III (400 MHz) instrument (Bruker, Berlin, Germany). Chemical shifts (δ, ppm) are presented relative to the trimethylsilane standard. Mass spectra were acquired using the Waters ZQ 2000 mass spectrometer (Waters, Milford, CT, USA). Thermogravimetric analysis (TGA) was carried out utilizing the TGAQ50 analyser (Verder Scientific Haan, Haan, Germany), while thermograms of differential scanning calorimetry (DSC) were recorded using the Bruker Reflex II DSC apparatus (Bruker, Berlin, Germany). For both types of thermal analysis, a heating rate of 10 °C/min in a nitrogen atmosphere was selected. Ultraviolet-visible (UV–vis) spectroscopy was performed using the HP-8453 diode array spectrometer (Agilent Technology Inc., Hachioji, Tokyo, Japan), and the resultant absorbance wavelengths were used to generate the Tauc plot. An Aminco-Bowman Series 2 spectrofluorometer (Agilent Technology Inc., Hachioji, Tokyo, Japan) were used to record photoluminescence (PL) spectra. Low-temperature PL (LTPL) spectra at 77 K to determine triplet energy was recorded with a Hitachi F-7000 fluorescence spectrophotometer (Edinburgh Instruments Ltd., Livingston, UK). The CH instrument CH1604A potentiostat (Annatech Co., Ltd., Taipei, Taiwan) was used to perform cyclic voltammetry (CV), and based on these results, HOMO levels were calculated. Time-resolved photoluminescence (TRPL) experiments, aiming to determine compound decay time, were conducted with an Edinburgh instrument FLS980 spectrometer (Edinburgh Instruments Ltd., Livingston, UK).

### 3.2. Synthesis and Structural Analysis

Carbazole (**1**), 1-bromooctane, 2-ethylhexylbromide, 1-bromohexane, 1-bromopentane, 1-bromobutane, bromoethane, FeCl_3_, KOH, K_2_CO_3_, Na_2_SO_4_, 4-fluorobenzophenone, chloroform, dimethyl sulfoxide (DMSO), and tetrahydrofuran (THF) were bought from Aldrich and used without further purification.

9H,9′H-3,3′-Bicarbazole (**2**), was synthesized using 9H-carbazole as a starting material and FeCl_3_ as an oxidising agent, as described earlier [68]. 

9-Ethyl-9′H-3,3′-bicarbazole (**3**) was synthesized by partially alkylating 9H,9′H-3,3′-bicarbazole (**2**), as it was described previously [69]. 

9-Butyl-9′H-3,3′-bicarbazole (**4**) was also synthesized by partially alkylating 9H,9′H-3,3′-bicarbazole (**2**) as it was described previously [69].

9-Pentyl-9′H-3,3′-bicarbazole (**5**). 9H,9′H-3,3′-bicarbazole (**2**) (2.00 g, 6.02 mmol) was dissolved in 50 mL of tetrahydrofuran, and 1-bromopentane (0.91 g, 6.02 mmol) was subsequently added. Potassium carbonate (1.66 g, 12.04 mmol) and powdered potassium hydroxide (2.02 g, 36.12 mmol) were gradually introduced while the solution was stirred continuously and heated to boiling temperature. After 4 h, TLC analysis was conducted, and the solution was filtered using a paper filter. The pure product was then isolated through column chromatography using tetrahydrofuran/hexane (volume ratio 1:5) as the mobile phase and silica gel as the stationary phase. The yield obtained was 1.06 g (44%) of pale-yellow material. ^1^H NMR (400 MHz, CDCl_3_, δ, m.d.): 8.47 (d, 2H, J = 10 Hz), 8.26 (d, 1H, 7.6 Hz), 8.23 (d, 1H, 8.0 Hz), 7.96 (s, 1H), 7.88 (d, 1H, J = 8.4 Hz), 7.84 (dd, 1H, J_1_ = 8.4 Hz, J_2_ = 1.6 Hz), 7.58–7.53 (m, 2H), 7.50–7.47 (m, 3H), 7.43 (d, 1H, J = 8 Hz), 7.33 (t, 2H, J = 7.2 Hz), 4.37 (t, 2H, J = 7.0 Hz), 1.96 (qu, 2H, J = 7.2 Hz), 1.49–1.41 (m, 4H), 0.96 (t, 3H, J = 7.2 Hz). ^13^C NMR (101 MHz, CDCl_3_, δ, m.d.): 140.98, 140.04, 139.65, 138.56, 133.33, 125.95, 125.87, 125.75, 125.60, 124.00, 123.61, 123.44, 123.10, 120.53, 120.47, 119.52, 119.02, 118.91, 118.83, 110.86, 110.79, 108.97, 108.87, 43.23, 29.50, 28.81, 22.58, 14.05.

9-Hexyl-9′H-3,3′-bicarbazole (**6**). 9H,9′H-3,3′-bicarbazole (**2**) (2.00 g, 6.02 mmol) was dissolved in 50 mL of tetrahydrofuran, and 1-bromohexane (0.99 g, 6.02 mmol) was subsequently added. Potassium carbonate (1.66 g, 12.04 mmol) and powdered potassium hydroxide (2.02 g, 36.12 mmol) were gradually introduced while the solution was stirred continuously and heated to boiling temperature. After 4 h, TLC analysis was conducted, and the solution was filtered using a paper filter. The pure product was then isolated through column chromatography using tetrahydrofuran/hexane (volume ratio 1:7) as the mobile phase and silica gel as the stationary phase. The yield obtained was 1.00 g (40%) of pale-yellow material. ^1^H NMR (400 MHz, CDCl_3_, δ, m.d.): 8.46 (d, 2H, J = 9.8 Hz), 8.25 (d, 1H, J = 8.0 Hz), 8.22 (d, 1H, J = 7.6 Hz), 7.99 (s, 1H), 7.88 (dd, 1H, J_1_ = 8.4 Hz, J_2_ = 1.6 Hz), 7.83 (dd, 1H, J_1_ = 8.4 Hz, J_2_ = 2.0 Hz), 7.57–7.43 (m, 6H), 7.32 (t, 2H, J = 7.4 Hz), 4.37 (t, 2H, J = 7.2 Hz), 1.95 (qu, 2H, J = 7.4 Hz), 1.50–1.33 (m, 6H), 0.94 (t, 3H, J = 7.2 Hz). ^13^C NMR (101 MHz, CDCl_3_, δ, m.d.): 140.97, 140.03, 139.64, 138.55, 134.13, 133.32, 125.95, 125.87, 125.73, 125.58, 124.00, 123.61, 123.44, 123.10, 120.51, 120.46, 119.51, 119.01, 118.91, 118.81, 110.83, 110.76, 108.95, 108.85, 43.26, 31.67, 29.05, 27.06, 22.62, 14.10.

9-(2-Ethylhexyl)-9′H-3,3′-bicarbazole (**7**) was synthesized by partially alkylating 9H,9′H-3,3′-bicarbazole (**2**), as it was described previously [67]. 

9-Octyl-9′H-3,3′-bicarbazole (**8**) was synthesized by partially alkylating 9H,9′H-3,3′-bicarbazole (**2**), as it was described previously [70]. 

4-(9′-Ethyl-[3,3′]-bicarbazol-9-yl)benzophenone (**DB41**) was synthesized by stirring 9-ethyl-9′H-3,3′-bicarbazole (**3**) (0.50 g, 1.39 mmol) with 4-fluorobenzophenone (0.28 g, 1.39 mmol) in 10 mL of DMSO at 150 °C under an inert nitrogen atmosphere with potassium carbonate (1.92 g, 13.90 mmol) present. After 4 h, TLC was used to confirm the completion of the reaction, following which the reaction mixture was slowly added to ice water. Chloroform was employed to extract the organic phase, and any remaining water traces in the organic phase were removed by adding anhydrous Na_2_SO_4_, which was filtered off later. The desired product was purified via column chromatography using tetrahydrofuran/hexane (volume ratio 1:3) as the mobile phase and silica gel as the stationary phase, resulting in a yellow amorphous material with a yield of 0.62 g (82%). T_g_ = 102 °C (DSC). ^1^H NMR (400 MHz, CDCl_3_, δ, m.d.): 8.48 (dd, 2H, J_1_ = 11.2 Hz, J_2_ = 1.6 Hz), 8.28 (d, 1H, J = 7.6 Hz), 8.24 (d, 1H, J = 7.6 Hz), 8.14 (d, 2H, J = 8.4 Hz), 7.97–7.95 (m, 1H), 7.89–7.81 (m, 4H), 7.69–7.65 (m, 2H), 7.61 (dd, 2H, J_1_ = 8.0 Hz, J_2_ = 1.6 Hz), 7.58–7.45 (m, 6H), 7.40 (t, 1H, J = 7.4 Hz), 7.33–7.30 (m, 1H), 4.46 (q, 2H, J = 7.2 Hz), 1.52 (t, 3H, J = 7.2 Hz). ^13^C NMR (101 MHz, CDCl_3_, δ, m.d.): 195.66, 141.81, 140.73, 140.46, 139.31, 139.21, 137.51, 135.97, 135.32, 132.95, 132.65, 131.95, 130.07, 128.49, 126.33, 126.21, 126.11, 125.81, 125.52, 124.51, 124.13, 123.59, 123.15, 120.69, 120.61, 120.56, 119.09, 119.00, 118.89, 110.07, 109.94, 108.73, 108.62, 37.69, 13.89. MS (APCI^+^, 20 V): 540.26 ([M + H], 100%).

4-(9′-Butyl-[3,3′]-bicarbazol-9-yl)benzophenone (**DB44**) was synthesized by stirring 9-butyl-9′H-3,3′-bicarbazole (**3**) (0.50 g, 1.29 mmol) with 4-fluorobenzophenone (0.26 g, 1.29 mmol) in 10 mL of DMSO at 150 °C under an inert nitrogen atmosphere with potassium carbonate (1.78 g, 12.90 mmol) present. After 4 h, TLC was used to confirm the completion of the reaction, following which the reaction mixture was slowly added to ice water. Chloroform was employed to extract the organic phase, and any remaining water traces in the organic phase were removed by adding anhydrous Na_2_SO_4_, which was filtered off later. The desired product was purified via column chromatography using tetrahydrofuran/hexane (volume ratio 1:5) as the mobile phase and silica gel as the stationary phase, resulting in a yellow amorphous material with a yield of 0.66 g (90%). T_g_ = 82 °C (DSC). ^1^H NMR (400 MHz, CDCl_3_, δ, m.d.): 8.49 (dd, 2H, J_1_ = 8.4 Hz, J_2_ = 1.6 Hz), 8.29 (d, 1H, J = 7.6 Hz), 8.25 (d, 1H, J = 7.6 Hz), 8.14 (d, 2H, J = 7.2 Hz), 7.97 (d, 2H, J = 7.2 Hz), 7.89–7.84 (m, 2H), 7.82 (d, 2H, 8.4 Hz), 7.69–7.65 (m, 2H), 7.63–7.58 (m, 3H), 7.56–7.47 (m, 4H), 7.40 (t, 1H, J = 7.4 Hz), 7.31 (t, 1H, J = 7.6 Hz), 4.39 (t, 2H, J = 7.2 Hz), 1.95 (qu, 2H, J = 7.4 Hz), 1.48 (sext, 2H, J = 7.6 Hz), 1.02 (t, 3H, J = 7.4 Hz). ^13^C NMR (101 MHz, CDCl_3_, δ, m.d.): 196.66, 141.81, 140.98, 140.73, 139.74, 139.31, 137.53, 135.96, 135.33, 132.89, 132.66, 131.96, 130.08, 128.50, 126.34, 126.21, 126.11, 125.78, 125.49, 124.52, 124.15, 123.48, 123.04, 120.71, 120.62, 120.50, 119.01, 118.86, 110.08, 109.96, 108.99, 108.87, 43.00, 31.23, 20.64, 13.96. MS (APCI^+^, 20 V): 568.39 ([M + H], 100%).

4-(9′-Pentyl-[3,3′]-bicarbazol-9-yl)benzophenone (**DB37**) was synthesized by stirring 9-pentyl-9′H-3,3′-bicarbazole (**3**) (0.50 g, 1.24 mmol) with 4-fluorobenzophenone (0.25 g, 1.24 mmol) in 10 mL of DMSO at 150 °C under an inert nitrogen atmosphere with potassium carbonate (1.72 g, 12.40 mmol) present. After 4 h, TLC was used to confirm the completion of the reaction, following which the reaction mixture was slowly added to ice water. Chloroform was employed to extract the organic phase, and any remaining water traces in the organic phase were removed by adding anhydrous Na_2_SO_4_, which was filtered off later. The desired product was purified via column chromatography using tetrahydrofuran/hexane (volume ratio 1:5) as the mobile phase and silica gel as the stationary phase, resulting in a yellow amorphous material with a yield of 0.66 g (91%). T_g_ = 77 °C (DSC). ^1^H NMR (400 MHz, CDCl_3_, δ, ppm): 8.47 (dd, 2H, J_1_ = 13.4 Hz, J_2_ = 1.4 Hz), 8.28 (d, 1H, J = 7.6 Hz), 8.23 (d, 1H, J = 7.6 Hz), 8.14 (d, 2H, J = 8.4 Hz), 7.97–7.94 (m, 2H), 7.88–7.81 (m, 4H), 7.69–7.65 (m, 2H), 7.62–7.57 (m, 3H), 7.55–7.46 (m, 4H), 7.39 (t, 1H, J = 7.4 Hz), 7.30 (t, 1H, J = 7.4 Hz), 4.38 (t, 2H, J = 7.2 Hz), 1.96 (qu, 2H, J = 7.2 Hz), 1.47–1.39 (m, 4H), 0.94 (t, 3H, J = 7.0 Hz). ^13^C NMR (101 MHz, CDCl_3_, δ, m.d.): 195.65, 141.80, 140.95, 140.71, 139.72, 139.29, 137.51, 135.95, 135.31, 132.86, 132.66, 131.96, 130.07, 128.49, 126.33, 126.20, 126.10, 125.78, 125.48, 124.50, 124.13, 123.46, 123.02, 120.69, 120.61, 120.48, 118.99, 118.84, 110.07, 109.94, 108.97, 108.85, 43.23, 29.49, 28.78, 22.54, 14.01. MS (APCI^+^, 20 V): 582.34 ([M + H], 100%).

4-(9′-Hexyl-[3,3′]-bicarbazol-9-yl)benzophenone (**DB38**) was synthesized by stirring 9-hexyl-9′H-3,3′-bicarbazole (**3**) (0.50 g, 1.20 mmol) with 4-fluorobenzophenone (0.24 g, 1.20 mmol) in 10 mL of DMSO at 150 °C under an inert nitrogen atmosphere with potassium carbonate (1.66 g, 12.00 mmol) present. After 4 h, TLC was used to confirm the completion of the reaction, following which the reaction mixture was slowly added to ice water. Chloroform was employed to extract the organic phase, and any remaining water traces in the organic phase were removed by adding anhydrous Na_2_SO_4_, which was filtered off later. The desired product was purified via column chromatography using tetrahydrofuran/hexane (volume ratio 1:7) as the mobile phase and silica gel as the stationary phase, resulting in a yellow amorphous material with a yield of 0.67 g (94%). T_g_ = 68 °C (DSC). ^1^H NMR (400 MHz, CDCl_3_, δ, ppm): 8.48 (dd, 2H, J_1_ = 12.4 Hz, J_2_ = 1.6 Hz), 8.28 (d, 1H, 7.6 Hz), 8.23 (d, 1H, J = 8.0 Hz), 8.14 (d, 2H, J = 8.8 Hz), 7.97–7.95 (m, 2H), 7.88–7.81 (m, 4H), 7.69–7.65 (m, 2H), 7.62–7.58 (m, 3H), 7.56–7.45 (m, 4H), 7.39 (t, 1H, J = 7.2 Hz), 7.30 (t, 1H, J = 7.2 Hz), 4.38 (t, 2H, J = 7.2 Hz), 1.95 (qu, 2H, J = 7.4 Hz), 1.49–1.33 (m, 6H), 0.92 (t, 3H, J = 7.0 Hz). ^13^C NMR (101 MHz, CDCl_3_-*d*_6_, δ, m.d.): 196.04, 141.80, 140.95, 140.70, 140.22, 139.01, 137.50, 135.95, 135.32, 132.86, 132.66, 131.97, 130.07, 128.55, 128.48, 127.24, 126.33, 126.21, 126.10, 125.77, 125.48, 124.49, 123.45, 123.01, 120.69, 120.62, 120.48, 119.00, 118.83, 110.07, 109.94, 108.97, 108.84, 43.26, 31.63, 29.03, 27.02, 22.60, 14.06. MS (APCI^+^, 20 V): 596.36 ([M + H], 100%).

4-(9′-{2-Ethylhexyl}-[3,3′]-bicarbazol-9-yl)benzophenone (**DB39**) was synthesized by stirring 9-{2-ethylhexyl}-9′H-3,3′-bicarbazole (**3**) (0.50 g, 1.13 mmol) with 4-fluorobenzophenone (0.23 g, 1.13 mmol) in 10 mL of DMSO at 150 °C under an inert nitrogen atmosphere with potassium carbonate (1.56 g, 11.30 mmol) present. After 4 h, TLC was used to confirm the completion of the reaction, following which the reaction mixture was slowly added to ice water. Chloroform was employed to extract the organic phase, and any remaining water traces in the organic phase were removed by adding anhydrous Na_2_SO_4_, which was filtered off later. The desired product was purified via column chromatography using tetrahydrofuran/hexane (volume ratio 1:7) as the mobile phase and silica gel as the stationary phase, resulting in a pale-yellow amorphous material with a yield of 0.56 g (79%). T_g_ = 64 °C (DSC). ^1^H NMR (400 MHz, CDCl_3_, δ, ppm): 8.48 (dd, 2H, J_1_ = 15.2 Hz, J_2_ = 1.6 Hz), 8.28 (d, 1H, J = 7.6 Hz), 8.24 (d, 1H, J = 7.6 Hz), 8.14 (d, 2H, J = 8.8 Hz), 7.97–7.95 (m, 2H), 7.86–7.81 (m, 4H), 7.69–7.65 (m, 2H), 7.62–7.58 (m, 3H), 7.55–7.45 (m, 4H), 7.40 (t, 1H, J = 7.2 Hz), 7.30 (t, 1H, J = 8.0 Hz), 4.26–4.23 (m, 2H), 2.21–2.13 (m, 1H), 1.50–1.31 (m, 8H), 0.98 (t, 3H, J = 7.4 Hz), 0.93 (t, 3H, J = 7.2 Hz). ^13^C NMR (101 MHz, CDCl_3_, δ, m.d.): 195.66, 141.81, 141.43, 140.71, 140.21, 139.29, 137.51, 135.94, 135.30, 132.82, 132.66, 131.97, 130.08, 128.50, 126.33, 126.20, 126.09, 125.75, 125.46, 124.51, 124.15, 123.42, 122.99, 120.70, 120.61, 120.43, 118.99, 118.92, 118.82, 110.08, 109.95, 109.27, 109.14, 47.57, 39.50, 31.07, 28.90, 24.46, 23.12, 14.11, 10.97. MS (APCI^+^, 20 V): 624.44 ([M + H], 100%).

4-(9′-Octyl-[3,3′]-bicarbazol-9-yl)benzophenone (**DB40**) was synthesized by stirring 9-octyl-9′H-3,3′-bicarbazole (**3**) (0.50 g, 1.13 mmol) with 4-fluorobenzophenone (0.23 g, 1.13 mmol) in 10 mL of DMSO at 150 °C under an inert nitrogen atmosphere with potassium carbonate (1.56 g, 11.30 mmol) present. After 4 h, TLC was used to confirm the completion of the reaction, following which the reaction mixture was slowly added to ice water. Chloroform was employed to extract the organic phase, and any remaining water traces in the organic phase were removed by adding anhydrous Na_2_SO_4_, which was filtered off later. The desired product was purified via column chromatography using tetrahydrofuran/hexane (volume ratio 1:7) as the mobile phase and silica gel as the stationary phase, resulting in a pale-yellow amorphous material with a yield of 0.67 g (95%). T_g_ = 57 °C (DSC). ^1^H NMR (400 MHz, CDCl_3_, δ, ppm): 8.48 (dd, 2H, J_1_ = 13.8 Hz, J_2_ = 1.4 Hz), 8.28 (d, 1H, J = 7.6 Hz), 8.24 (d, 1H, J = 7.6 Hz), 8.14 (d, 2H, J = 8.4 Hz), 7.97–7.95 (m, 2H), 7.86–7.81 (m, 4H), 7.70–7.65 (m, 2H), 7.62–7.58 (m, 3H), 7.56–7.46 (m, 4H), 7.39 (t, 1H, J = 7.4 Hz), 7.30 (t, 1H, J = 7.6 Hz), 4.38 (t, 2H, J = 7.6 Hz), 1.95 (pent, 2H, J = 7.6 Hz), 1.48–1.29 (m, 10H), 0.91 (t, 3H, J = 7.4 Hz). ^13^C NMR (101 MHz, CDCl_3_, δ, m.d.): 195.66, 141.81, 140.96, 140.72, 139.72, 139.30, 137.51, 135.96, 135.33, 132.87, 132.65, 131.95, 130.07, 128.49, 126.32, 126.21, 126.10, 125.77, 125.48, 124.50, 124.14, 123.46, 123.02, 120.69, 120.60, 120.48, 119.00, 118.83, 110.06, 109.94, 108.97, 108.85, 43.26, 31.83, 29.43, 29.21, 29.06, 27.37, 22.63, 14.09. MS (APCI^+^, 20 V): 624.44 ([M + H], 100%).

### 3.3. Fabrication and Characterization of Devices

A glass substrate with a pre-patterned indium tin oxide (ITO) layer, bought from Lumtec (Taiwan), was utilized in the production process of OLEDs. The mentioned substrate underwent a cleaning procedure with acetone for 30 min at 50 °C, followed by a cleaning with isopropyl alcohol for 30 min at 60 °C. Subsequently, the cleaned substrates were exposed to UV radiation for 10 min in a preheated UV chamber. The layer deposition process took place within a glove box under an inert atmosphere. For the next layer, the hole-injecting material poly(3,4-ethylene-dioxythiophene):poly(styrenesulfonate) (PEDOT:PSS) was employed. It was spin-coated onto the substrate at 4000 rpm for 20 s, followed by heating the substrates for 10 min at 130 °C. After cooling the substrates, the emissive layers, composed of host material and emitter, were formed on top of the hole injection layer. For deposition, spin-coating was also employed by spinning substrates for 20 s at 2500 rpm. Subsequently, the electron transporting layer, consisting of 1,3,5-tris(N-phenyl-benzimidazol-2-yl)benzene (TPBi), a LiF electron injecting layer, and aluminum cathode, were formed in a thermal evaporation chamber under a vacuum of 10^−6^ torr. Following this, the resulting devices, with an area of 0.09 cm², were placed in a mini chamber within the glove box under vacuum until further tests were conducted. These tests were performed under normal atmospheric conditions in complete darkness. The CS-100A luminance and color meter (Konica Minolta, Tokyo, Japan) was utilized to record voltage-current density and voltage-luminance characteristics. Additionally, the SpectraScan^®^ spectroradiometer PR-655 (Jadak, North Syracuse, NY, USA) was used to create luminance-power efficacy and luminance-current graphs. The measurements of current-voltage characteristics were conducted using a Keithley voltmeter (Keithley Instruments, Cleveland, OH, USA). EQE was calculated using the method outlined in the literature [71]. 

## 4. Conclusions

We introduced novel emissive derivatives synthesized through a three-step process, utilizing bicarbazole and benzophenone as building blocks to achieve twisted donor-acceptor structures. The incorporation of alkyl sidechains of varying length was chosen to enhance the solubility and film-forming characteristics of the materials. Newly synthesized derivatives exhibited commendable thermal and morphological stability, as evidenced by temperatures of 5% mass loss ranging from 374 °C to 406 °C. The manipulation of alkyl sidechain length allowed control over glass-transition temperatures that spanned from 57 °C to a desirable 102 °C. Additionally, newly developed materials demonstrated short photoluminescence decay times, confirmed by time-resolved photoluminescence, and high photoluminescence quantum yields of up to 75.5%. The benzophenone-based derivatives exhibited favourable HOMO-LUMO levels as well as suitable triplet-singlet state energy values for application as potential blue TADF emitters. Upon investigation of the electroluminescent properties of the new devices, an OLED with an emissive layer comprised of 15 wt% **DB39** doped in a CBP host surpassed other devices in terms of efficiencies. The maximum current efficacy (CE_max_) reaching 5.7 cd/A and 2.7% external quantum efficacy (EQE_max_) were detected, followed by a maximum luminance (L_max_) of 3581 cd/m^2^ with a turn-on voltage of 3.9 V. This study emphasized the notable influence of energy transfer from host to guest, suitable doping concentrations, and the effect of chemical structure on solubility, thereby affecting the efficiency of wet-processed devices. It is crucial to highlight that these characteristics were observed in non-optimized OLEDs using standard laboratory conditions, suggesting potential enhancements through optimization processes. Furthermore, enhanced device efficiency could be achieved by reducing ∆E_ST_ and effectively utilizing triplet-state excitons of similar materials, making them suitable for highly efficient lighting applications. We believe that our findings suggest the potential of some materials for further exploration as promising emitters.

## Figures and Tables

**Table 1:** Characteristics of the derivatives **DB37**, **DB38**, **DB39**, **DB40**, **DB41** and **DB44**.

Emitter	λ_ex_ (nm)	λ_em_ (nm)	Φ (%)	Homo (eV)	Lumo (eV)	E_g_ (eV)	Decay (ns)	S_1_ (eV)	T_1_ (eV)	ΔE_ST_	T_d_ (°C)	T_g_ (°C)
**DB37**	384.5, 395.6	509	65.5	−5.67	−2.58	3.09	5.53	3.04	2.76	0.28	406	102
**DB38**	383.5, 400	510	45.3	−5.70	−2.61	3.09	1.88	2.94	2.89	0.05	398	80
**DB39**	382.7, 400	528	75.5	−5.68	−2.60	3.08	4.27	3.10	2.81	0.29	383	77
**DB40**	384.5, 408.2	513	52.5	−5.69	−2.59	3.10	2.41	3.06	2.80	0.26	397	68
**DB41**	381.0, 399.7	528	62.5	−5.73	−2.64	3.09	2.24	3.22	2.80	0.42	374	64
**DB44**	383.9, 398.6	529	68.5	−5.69	−2.62	3.07	6.28	3.18	2.82	0.15	389	57

λ_ex_: Excitation Wavelength; λ_em_: Emission Wavelength; Φ: Photoluminescence Yield; E_g_: Bandgap S_1_: Singlet Energy; T_1_: Triplet Energy; ΔE_ST_: Singlet-Triplet Energy Gap; T_d_: Destruction temperature; T_g_: Glass Transition Temperature.

**Table 2:** EL characteristics of the OLEDs containing emitting materials **DB37**, **DB38**, **DB39**, **DB40**, **DB41**, and **DB44**.

Emitter	Concentration (wt%)	Turn-On Voltage (Von) ^a^	Power Efficacy (lm/W)	Current Efficacy (cd/A)	EQE (%)	CIE_xy_	L_Max_ (cd/m^2^)
			@100 cd/m^2^/@1000 cd/m^2^/max			@100 cd/m^2^/@1000 cd/m^2^	
**DB37**	5.0	4.0	2.1/1.1/3.4	3.4/2.4/3.9	2.1/1.6/2.1	(0.17, 0.22)/(0.17, 0.30)	3449
	10	3.5	2.5/1.3/3.4	3.5/2.5/3.8	1.8/1.5/1.9	(0.18, 0.26)/(0.18, 0.23)	3658
	15	3.4	2.8/1.5/3.6	3.7/2.7/4.0	1.7/1.5/1.8	(0.19, 0.28)/(0.18, 0.25)	3464
	100	3.1	0.3/-/-	0.3/-/-	0.1/-/-	(0.24, 0.40)/-	616
**DB38**	5.0	3.9	1.9/1.0/3.4	3.1/2.2/3.8	1.9/1.5/1.9	(0.18, 0.22)/(0.17, 0.20)	2801
	10	3.5	2.7/1.4/2.9	3.7/2.7/3.8	2.0/1.6/1.9	(0.18, 0.25)/(0.18, 0.22)	3430
	15	3.4	2.8/1.5/3.5	3.6/2.8/4.2	1.7/1.6/1.8	(0.19, 0.27)/(0.18, 0.24)	3555
	100	3.2	0.2/-/-	0.3/-/-	0.1/-/-	(0.22, 0.38)/-	618
**DB39**	5.0	4.0	1.8/0.9/3.3	3.1/2.2/3.7	2.0/1.6/2.1	(0.18, 0.20)/(0.17, 0.18)	2818
	10	3.5	2.5/1.3/4.4	3.5/2.7/4.9	2.0/1.8/2.2	(0.18, 0.23)/(0.18, 0.21)	3430
	15	3.9	3.0/1.4/4.1	4.4/2.8/5.7	2.2/1.6/2.7	(0.19, 0.27)/(0.19, 0.24)	3581
	100	3.4	0.3/-/-	0.4/-/-	0.4/-/-	(0.24, 0.39)/-	615
**DB40**	5.0	4.2	2.0/1.1/2.1	3.4/2.4/3.4	2.2/1.6/2.3	(0.17, 0.22)/(0.17, 0.20)	3166
	10	3.5	2.8/1.5/2.9	3.8/2.8/3.7	2.0/1.7/2.0	(0.18, 0.25)/(0.18, 0.22)	3840
	15	3.3	2.8/1.6/2/8	3.6/2.8/3.6	1.8/1.6/1.8	(0.18, 0.27)/(0.18, 0.24)	3950
	100	3.2	0.2/-/-	0.3/-/-	0.1/-/-	(0.22, 0.38)/-	685
**DB41**	5.0	4.4	1.6/0.8/1.9	2.9/1.9/3.1	1.9/1.3/2.0	(0.18, 0.21)/(0.18, 0.19)	2687
	10	3.8	2.4/1.1/2.4	3.6/2.5/3.6	2.0/1.5/2.0	(0.19, 0.26)/(0.19, 0.23)	3347
	15	3.5	2.6/1.3/2.7	3.6/2.5/3.6	1.7/1.1/1.8	(0.20, 0.28)/(0.19, 0.24)	3128
	100	3.1	0.2/-/-	0.3/-/-	0.1/-/-	(0.30, 0.45)/-	486
**DB44**	5.0	4.8	1.6/0.7/1.6	3.3/2.2/3.3	2.3/-/2.3	(0.17, 0.20)/-	1718
	10	4.0	2.1/0.8/2.7	3.9/2.3/4.1	2.1/1.4/2.3	(0.18, 0.24)/(0.18, 0.22)	1283
	15	3.8	2.1/0.5/2.3	3.6/1.5/3.7	1.9/-/2.0	(0.19, 0.26)/-	1275
	100	5.1	-/-/-	-/-/-	-/-/-	-/-	55

^a^ Turn-on voltage at luminance > 1 cd/m^2^. Characteristics of best-performing device are highlighted in grey.

## Data Availability

The data presented in this study are available on request from the corresponding authors.

## Supplementary Materials

The following supporting information can be downloaded at: https://www.mdpi.com/article/10.3390/molecules29071672/s1. Figure S1: TGA curves of materials **DB37**, **DB38**, **DB39**, **DB40**, **DB41**, and **DB44**, Figure S2: UV-Vis absorption bands and Tauc plots of materials **DB37**, **DB38**, **DB39**, **DB40**, **DB41**, and **DB44**, Figure S3: LTPL spectra at 77 K and triplet energy calculation of the derivatives **DB37**, **DB38**, **DB39**, **DB40**, **DB41**, and **DB44**, Figures S4–S8: The electroluminescent (EL) characteristics of OLEDs with dopants **DB37**, **DB38**, **DB40**, **DB41**, and **DB44**, respectively, doped within a CBP host matrix at different concentrations, displaying EL spectra, current density–voltage, luminance–voltage, power efficacy–luminance, and current efficacy–luminance dependencies.

## References

[1] Hong G., Gan X., Leonhardt C., Zhang Z., Seibert J., Busch J.M., Bräse S. (2021). A Brief History of OLEDs—Emitter Development and Industry Milestones. Adv. Mater..

[2] Luo Y.-J., Lu Z.-Y., Huang Y. (2016). Triplet Fusion Delayed Fluorescence Materials for OLEDs. Chin. Chem. Lett..

[3] Zeng H., Huang Q., Liu J., Huang Y., Zhou J., Zhao S., Lu Z. (2016). A Red-Emissive Sextuple Hydrogen-Bonding Self-Assembly Molecular Duplex Bearing Perylene Diimide Fluorophores for Warm-White Organic Light-Emitting Diode Application. Chin. J. Chem..

[4] Zhang Z., Li W., Ye K., Zhang H. (2016). Synthesis, Structure and Properties of a Novel Benzothiazole-Based Diboron-Bridged π-Conjugated Ladder. Acta Chim. Sin..

[5] Yu Y., Yang J., Ren Z., Xie G., Li Q., Li Z. (2016). Synthesis of Solution Processable Blue AIEgens and the Device Performance. Acta Chim. Sin..

[6] Im Y., Byun S.Y., Kim J.H., Lee D.R., Oh C.S., Yook K.S., Lee J.Y. (2017). Recent Progress in High-Efficiency Blue-Light-Emitting Materials for Organic Light-Emitting Diodes. Adv. Funct. Mater..

[7] Root S.E., Savagatrup S., Printz A.D., Rodriquez D., Lipomi D.J. (2017). Mechanical Properties of Organic Semiconductors for Stretchable, Highly Flexible, and Mechanically Robust Electronics. Chem. Rev..

[8] Kim J.-J., Han M.-K., Noh Y.-Y. (2011). Flexible OLEDs and Organic Electronics. Semicond. Sci. Technol..

[9] Jeong E.G., Kwon J.H., Kang K.S., Jeong S.Y., Choi K.C. (2020). A Review of Highly Reliable Flexible Encapsulation Technologies towards Rollable and Foldable OLEDs. J. Inf. Disp..

[10] Reineke S., Lindner F., Schwartz G., Seidler N., Walzer K., Lüssem B., Leo K. (2009). White Organic Light-Emitting Diodes with Fluorescent Tube Efficiency. Nature.

[11] Sun Y., Giebink N.C., Kanno H., Ma B., Thompson M.E., Forrest S.R. (2006). Management of Singlet and Triplet Excitons for Efficient White Organic Light-Emitting Devices. Nature.

[12] Wang J., Liang J., Xu Y., Liang B., Wei J., Li C., Mu X., Ye K., Wang Y. (2019). Purely Organic Phosphorescence Emitter-Based Efficient Electroluminescence Devices. J. Phys. Chem. Lett..

[13] Yang X., Yue L., Yu Y., Liu B., Dang J., Sun Y., Zhou G., Wu Z., Wong W. (2020). Strategically Formulating Aggregation-Induced Emission-Active Phosphorescent Emitters by Restricting the Coordination Skeletal Deformation of Pt(II) Complexes Containing Two Independent Monodentate Ligands. Adv. Opt. Mater..

[14] Rajamalli P., Senthilkumar N., Huang P.-Y., Ren-Wu C.-C., Lin H.-W., Cheng C.-H. (2017). New Molecular Design Concurrently Providing Superior Pure Blue, Thermally Activated Delayed Fluorescence and Optical Out-Coupling Efficiencies. J. Am. Chem. Soc..

[15] Zhu M., Yang C. (2013). Blue Fluorescent Emitters: Design Tactics and Applications in Organic Light-Emitting Diodes. Chem. Soc. Rev..

[16] Du C., Lu T., Cheng Z., Chang Y., Liu H., Wang J., Wan L., Lv Y., Lu P. (2022). Rational Molecular Design of Phenanthroimidazole-Based Fluorescent Materials towards High-Efficiency Non-Doped Deep Blue OLEDs. J. Mater. Chem. C Mater..

[17] Xu H., Chen R., Sun Q., Lai W., Su Q., Huang W., Liu X. (2014). Recent Progress in Metal–Organic Complexes for Optoelectronic Applications. Chem. Soc. Rev..

[18] De Leeuw D.M., Simenon M.M.J., Brown A.R., Einerhand R.E.F. (1997). Stability of N-Type Doped Conducting Polymers and Consequences for Polymeric Microelectronic Devices. Synth. Met..

[19] Scholz S., Kondakov D., Lüssem B., Leo K. (2015). Degradation Mechanisms and Reactions in Organic Light-Emitting Devices. Chem. Rev..

[20] Lee J., Jeong C., Batagoda T., Coburn C., Thompson M.E., Forrest S.R. (2017). Hot Excited State Management for Long-Lived Blue Phosphorescent Organic Light-Emitting Diodes. Nat. Commun..

[21] Xing L., Zhu Z.-L., He J., Qiu Z., Yang Z., Lin D., Chen W.-C., Yang Q., Ji S., Huo Y. (2021). Anthracene-Based Fluorescent Emitters toward Superior-Efficiency Nondoped TTA-OLEDs with Deep Blue Emission and Low Efficiency Roll-Off. Chem. Eng. J..

[22] Chen J., Tao W., Chen W., Xiao Y., Wang K., Cao C., Yu J., Li S., Geng F., Adachi C. (2019). Red/Near-Infrared Thermally Activated Delayed Fluorescence OLEDs with Near 100% Internal Quantum Efficiency. Angew. Chem. Int. Ed..

[23] Albrecht K., Matsuoka K., Fujita K., Yamamoto K. (2015). Carbazole Dendrimers as Solution-Processable Thermally Activated Delayed-Fluorescence Materials. Angew. Chem. Int. Ed..

[24] Zhang Q., Li J., Shizu K., Huang S., Hirata S., Miyazaki H., Adachi C. (2012). Design of Efficient Thermally Activated Delayed Fluorescence Materials for Pure Blue Organic Light Emitting Diodes. J. Am. Chem. Soc..

[25] Wu K., Zhang T., Wang Z., Wang L., Zhan L., Gong S., Zhong C., Lu Z.-H., Zhang S., Yang C. (2018). De Novo Design of Excited-State Intramolecular Proton Transfer Emitters via a Thermally Activated Delayed Fluorescence Channel. J. Am. Chem. Soc..

[26] Goushi K., Yoshida K., Sato K., Adachi C. (2012). Organic Light-Emitting Diodes Employing Efficient Reverse Intersystem Crossing for Triplet-to-Singlet State Conversion. Nat. Photonics.

[27] Ahn D.H., Kim S.W., Lee H., Ko I.J., Karthik D., Lee J.Y., Kwon J.H. (2019). Highly Efficient Blue Thermally Activated Delayed Fluorescence Emitters Based on Symmetrical and Rigid Oxygen-Bridged Boron Acceptors. Nat. Photonics.

[28] Uoyama H., Goushi K., Shizu K., Nomura H., Adachi C. (2012). Highly Efficient Organic Light-Emitting Diodes from Delayed Fluorescence. Nature.

[29] Wang Z., Li Y., Cai X., Chen D., Xie G., Liu K., Wu Y.-C., Lo C.-C., Lien A., Cao Y. (2016). Structure–Performance Investigation of Thioxanthone Derivatives for Developing Color Tunable Highly Efficient Thermally Activated Delayed Fluorescence Emitters. ACS Appl. Mater. Interfaces.

[30] Im Y., Kim M., Cho Y.J., Seo J.-A., Yook K.S., Lee J.Y. (2017). Molecular Design Strategy of Organic Thermally Activated Delayed Fluorescence Emitters. Chem. Mater..

[31] Cai X., Li X., Xie G., He Z., Gao K., Liu K., Chen D., Cao Y., Su S.-J. (2016). “Rate-Limited Effect” of Reverse Intersystem Crossing Process: The Key for Tuning Thermally Activated Delayed Fluorescence Lifetime and Efficiency Roll-off of Organic Light Emitting Diodes. Chem. Sci..

[32] Hatakeyama T., Shiren K., Nakajima K., Nomura S., Nakatsuka S., Kinoshita K., Ni J., Ono Y., Ikuta T. (2016). Ultrapure Blue Thermally Activated Delayed Fluorescence Molecules: Efficient HOMO–LUMO Separation by the Multiple Resonance Effect. Adv. Mater..

[33] Zheng X., Huang R., Zhong C., Xie G., Ning W., Huang M., Ni F., Dias F.B., Yang C. (2020). Achieving 21% External Quantum Efficiency for Nondoped Solution-Processed Sky-Blue Thermally Activated Delayed Fluorescence OLEDs by Means of Multi-(Donor/Acceptor) Emitter with Through-Space/-Bond Charge Transfer. Adv. Sci..

[34] Ma F., Ji H., Zhang D., Xue K., Zhang P., Qi Z., Zhu H. (2021). Adjusting the Photophysical Properties of AIE-Active TADF Emitters from through-Bond to through-Space Charge Transfer for High-Performance Solution-Processed OLEDs. Dye. Pigment..

[35] Rajamalli P., Rota Martir D., Zysman-Colman E. (2018). Pyridine-Functionalized Carbazole Donor and Benzophenone Acceptor Design for Thermally Activated Delayed Fluorescence Emitters in Blue Organic Light-Emitting Diodes. J. Photonics Energy.

[36] Ma M., Li J., Liu D., Li D., Dong R., Mei Y. (2021). Low Efficiency Roll-off Thermally Activated Delayed Fluorescence Emitters for Non-Doped OLEDs: Substitution Effect of Thioether and Sulfone Groups. Dye. Pigment..

[37] Wu L., Wang K., Wang C., Fan X.-C., Shi Y.-Z., Zhang X., Zhang S.-L., Ye J., Zheng C.-J., Li Y.-Q. (2021). Using Fluorene to Lock Electronically Active Moieties in Thermally Activated Delayed Fluorescence Emitters for High-Performance Non-Doped Organic Light-Emitting Diodes with Suppressed Roll-Off. Chem. Sci..

[38] Aizawa N., Tsou C.-J., Park I.S., Yasuda T. (2017). Aggregation-Induced Delayed Fluorescence from Phenothiazine-Containing Donor–Acceptor Molecules for High-Efficiency Non-Doped Organic Light-Emitting Diodes. Polym. J..

[39] Jing Y.-Y., Tao X.-D., Yang M.-X., Chen X.-L., Lu C.-Z. (2021). Triptycene-Imbedded Thermally Activated Delayed Fluorescence Emitters with Excellent Film Morphologies for Applications in Efficient Nondoped and Doped Organic Light-Emitting Devices. Chem. Eng. J..

[40] Tani K., Yashima T., Miyanaga K., Hori K., Goto K., Tani F., Habuka Y., Suzuki K., Shizu K., Kaji H. (2018). Carbazole and Benzophenone Based Twisted Donor–Acceptor Systems as Solution Processable Green Thermally Activated Delayed Fluorescence Organic Light Emitters. Chem. Lett..

[41] Liu Y., Wu X., Chen Y., Chen L., Li H., Wang W., Wang S., Tian H., Tong H., Wang L. (2019). Triazatruxene-Based Thermally Activated Delayed Fluorescence Small Molecules with Aggregation-Induced Emission Properties for Solution-Processable Nondoped OLEDs with Low Efficiency Roll-Off. J. Mater. Chem. C Mater..

[42] Wang J., Zhang J., Jiang C., Yao C., Xi X. (2021). Effective Design Strategy for Aggregation-Induced Emission and Thermally Activated Delayed Fluorescence Emitters Achieving 18% External Quantum Efficiency Pure-Blue OLEDs with Extremely Low Roll-Off. ACS Appl. Mater. Interfaces.

[43] Shizu K., Lee J., Tanaka H., Nomura H., Yasuda T., Kaji H., Adachi C. (2015). Highly Efficient Electroluminescence from Purely Organic Donor–Acceptor Systems. Pure Appl. Chem..

[44] Nishimoto T., Yasuda T., Lee S.Y., Kondo R., Adachi C. (2014). A Six-Carbazole-Decorated Cyclophosphazene as a Host with High Triplet Energy to Realize Efficient Delayed-Fluorescence OLEDs. Mater. Horiz..

[45] Huang B., Ban X., Sun K., Ma Z., Mei Y., Jiang W., Lin B., Sun Y. (2016). Thermally Activated Delayed Fluorescence Materials Based on Benzophenone Derivative as Emitter for Efficient Solution-Processed Non-Doped Green OLED. Dye. Pigment..

[46] Liang J., Li C., Zhuang X., Ye K., Liu Y., Wang Y. (2018). Novel Blue Bipolar Thermally Activated Delayed Fluorescence Material as Host Emitter for High-Efficiency Hybrid Warm-White OLEDs with Stable High Color-Rendering Index. Adv. Funct. Mater..

[47] Pocock I.A., Alotaibi A.M., Jagdev K., Prior C., Burgess G.R., Male L., Grainger R.S. (2021). Direct Formation of 4,5-Disubstituted Carbazoles via Regioselective Dilithiation. Chem. Commun..

[48] Zhu X.-D., Tian Q.-S., Zheng Q., Tao X.-C., Yuan Y., Yu Y.-J., Li Y., Jiang Z.-Q., Liao L.-S. (2019). A Sky-Blue Thermally Activated Delayed Fluorescence Emitter Based on Multimodified Carbazole Donor for Efficient Organic Light-Emitting Diodes. Org. Electron..

[49] Liu F., Zou J., He Q., Tang C., Xie L., Peng B., Wei W., Cao Y., Huang W. (2010). Carbazole End-capped Pyrene Starburst with Enhanced Electrochemical Stability and Device Performance. J. Polym. Sci. A Polym. Chem..

[50] Sęk D., Szlapa-Kula A., Siwy M., Fabiańczyk A., Janeczek H., Szalkowski M., Maćkowski S., Schab-Balcerzak E. (2020). Branched Azomethines Based on Tris(2-Aminoethyl)Amine: Impact of Imine Core Functionalization on Thermal, Electrochemical and Luminescence Properties. Mater. Chem. Phys..

[51] Sebris A., Novosjolova I., Traskovskis K., Kokars V., Tetervenoka N., Vembris A., Turks M. (2022). Photophysical and Electrical Properties of Highly Luminescent 2/6-Triazolyl-Substituted Push–Pull Purines. ACS Omega.

[52] Yang Z., Chi Z., Yu T., Zhang X., Chen M., Xu B., Liu S., Zhang Y., Xu J. (2009). Triphenylethylene Carbazole Derivatives as a New Class of AIE Materials with Strong Blue Light Emission and High Glass Transition Temperature. J. Mater. Chem..

[53] Costa J.C.S., Lima M.A.L., Mendes A., Santos L.M.N.B.F. (2020). The Impact of Phenyl–Phenyl Linkage on the Thermodynamic, Optical and Morphological Behavior of Carbazol Derivatives. RSC Adv..

[54] Huang T., Jiang W., Duan L. (2018). Recent Progress in Solution Processable TADF Materials for Organic Light-Emitting Diodes. J. Mater. Chem. C Mater..

[55] Wang J., Liu C., Jiang C., Yao C., Gu M., Wang W. (2019). Solution-Processed Aggregation-Induced Delayed Fluorescence (AIDF) Emitters Based on Strong π-Accepting Triazine Cores for Highly Efficient Nondoped OLEDs with Low Efficiency Roll-Off. Org. Electron..

[56] Li Y., Xie G., Gong S., Wu K., Yang C. (2016). Dendronized Delayed Fluorescence Emitters for Non-Doped, Solution-Processed Organic Light-Emitting Diodes with High Efficiency and Low Efficiency Roll-off Simultaneously: Two Parallel Emissive Channels. Chem. Sci..

[57] Sun S., Wang J., Chen L., Chen R., Jin J., Chen C., Chen S., Xie G., Zheng C., Huang W. (2019). Thermally Activated Delayed Fluorescence Enantiomers for Solution-Processed Circularly Polarized Electroluminescence. J. Mater. Chem. C Mater..

[58] Inoue S., Minemawari H., Tsutsumi J., Chikamatsu M., Yamada T., Horiuchi S., Tanaka M., Kumai R., Yoneya M., Hasegawa T. (2015). Effects of Substituted Alkyl Chain Length on Solution-Processable Layered Organic Semiconductor Crystals. Chem. Mater..

[59] Schmaljohann D., Häußler L., Pötschke P., Voit B.I., Loontjens T.J.A. (2000). Modification with Alkyl Chains and the Influence on Thermal and Mechanical Properties of Aromatic Hyperbranched Polyesters. Macromol. Chem. Phys..

[60] Makuła P., Pacia M., Macyk W. (2018). How To Correctly Determine the Band Gap Energy of Modified Semiconductor Photocatalysts Based on UV–Vis Spectra. J. Phys. Chem. Lett..

[61] Zhao L., Liu Y., Wang S., Tao Y., Wang F., Zhang X., Huang W. (2017). Novel Hyperbranched Polymers as Host Materials for Green Thermally Activated Delayed Fluorescence OLEDs. Chin. J. Polym. Sci..

[62] Sworakowski J. (2018). How Accurate Are Energies of HOMO and LUMO Levels in Small-Molecule Organic Semiconductors Determined from Cyclic Voltammetry or Optical Spectroscopy?. Synth. Met..

[63] Li F., Gillett A.J., Gu Q., Ding J., Chen Z., Hele T.J.H., Myers W.K., Friend R.H., Evans E.W. (2022). Singlet and Triplet to Doublet Energy Transfer: Improving Organic Light-Emitting Diodes with Radicals. Nat. Commun..

[64] Chen C., Huang R., Batsanov A.S., Pander P., Hsu Y., Chi Z., Dias F.B., Bryce M.R. (2018). Intramolecular Charge Transfer Controls Switching Between Room Temperature Phosphorescence and Thermally Activated Delayed Fluorescence. Angew. Chem. Int. Ed..

[65] Liu X., Lv D., Wang S., Yu X., Han Y. (2024). Improving Film Uniformity and Interface Solvent Resistance to Realize Multilayer Printing of OLED Devices. J. Mater. Chem. C Mater..

[66] Wang G., Chernikov A., Glazov M.M., Heinz T.F., Marie X., Amand T., Urbaszek B. (2018). *Colloquium*: Excitons in Atomically Thin Transition Metal Dichalcogenides. Rev. Mod. Phys..

[67] Trovatello C., Katsch F., Borys N.J., Selig M., Yao K., Borrego-Varillas R., Scotognella F., Kriegel I., Yan A., Zettl A. (2020). The Ultrafast Onset of Exciton Formation in 2D Semiconductors. Nat. Commun..

[68] Vaitkeviciene V., Grigalevicius S., Grazulevicius J.V., Jankauskas V., Syromyatnikov V.G. (2006). Hole-Transporting [3,3′]Bicarbazolyl-Based Polymers and Well-Defined Model Compounds. Eur. Polym. J..

[69] Gautam P., Shahnawaz, Siddiqui I., Blazevicius D., Krucaite G., Tavgeniene D., Jou J.-H., Grigalevicius S. (2023). Bifunctional Bicarbazole-Benzophenone-Based Twisted Donor–Acceptor–Donor Derivatives for Deep-Blue and Green OLEDs. Nanomaterials.

[70] Blazevicius D., Siddiqui I., Gautam P., Krucaite G., Tavgeniene D., Nagar M.R., Kumar K., Banik S., Jou J.-H., Grigalevicius S. (2024). Bicarbazole-Benzophenone-Based Twisted Donor-Acceptor-Donor Derivatives as Blue Emitters for Highly Efficient Fluorescent Organic Light-Emitting Diodes. Nanomaterials.

[71] De Sa Pereira D., Data P., Monkman A.P. (2017). Methods of Analysis of Organic Light Emitting Diodes. Display.

