# Supplementary material for: Bicarbazole-Benzophenone Based Twisted Donor-Acceptor Derivatives as Potential Blue TADF Emitters for OLEDs
Source: Molecules. 2024 Apr 8;29(7):1672. doi: 10.3390/molecules29071672 (PMC11013760; doi:10.3390/molecules29071672)
Supplement: Supplementary file 1 [file molecules-29-01672-s001.zip › molecules-2909825-supplementary.pdf]

Supplementary material

Bicarbazole-benzophenone based twisted donor-acceptor derivatives as potential blue TADF emitters for OLEDs

Iram Siddiqui[1,§], Prakalp Gautam[1,§], Dovydas Blazevicius[2,§], Jayakumar Jayachandran[1], Sushanta Lenka[1], Daiva Tavgeniene[2], Ernestas Zaleckas[3], Saulius Grigalevicius*[2] and Jwo-Huei Jou*[1]

[1] Department of Materials Science and Engineering, National Tsing Hua University, Hsinchu 30044, Taiwan; jayakumar@mx.nthu.edu.tw (J.J.); sushantalenka1@gmail.com (S.L.)

[2] Department of Polymer Chemistry and Technology, Kaunas University of Technology, Radvilenu plentas 19, Kaunas, LT50254, Lithuania; daiva.tavgeniene@ktu.lt (D.T.)

[3] Vytautas Magnus University, Agriculture Academy, Department of Agricultural Engineering and Safety, Studentu str. 11, LT-53361, Akademija, Kaunas distr., Lithuania

* Correspondence: saulius.grigalevicius@ktu.lt (S.G.); jjou@mx.nthu.edu.tw (J.-H.J.)

§ These authors contributed equally to this work.

Table of Contents

1. Thermogravimetric analysis
2. Ultraviolet-visible absorption bands and Tauc plots
3. Triplet energy
4. Electroluminescence

1. Thermogravimetric analysis

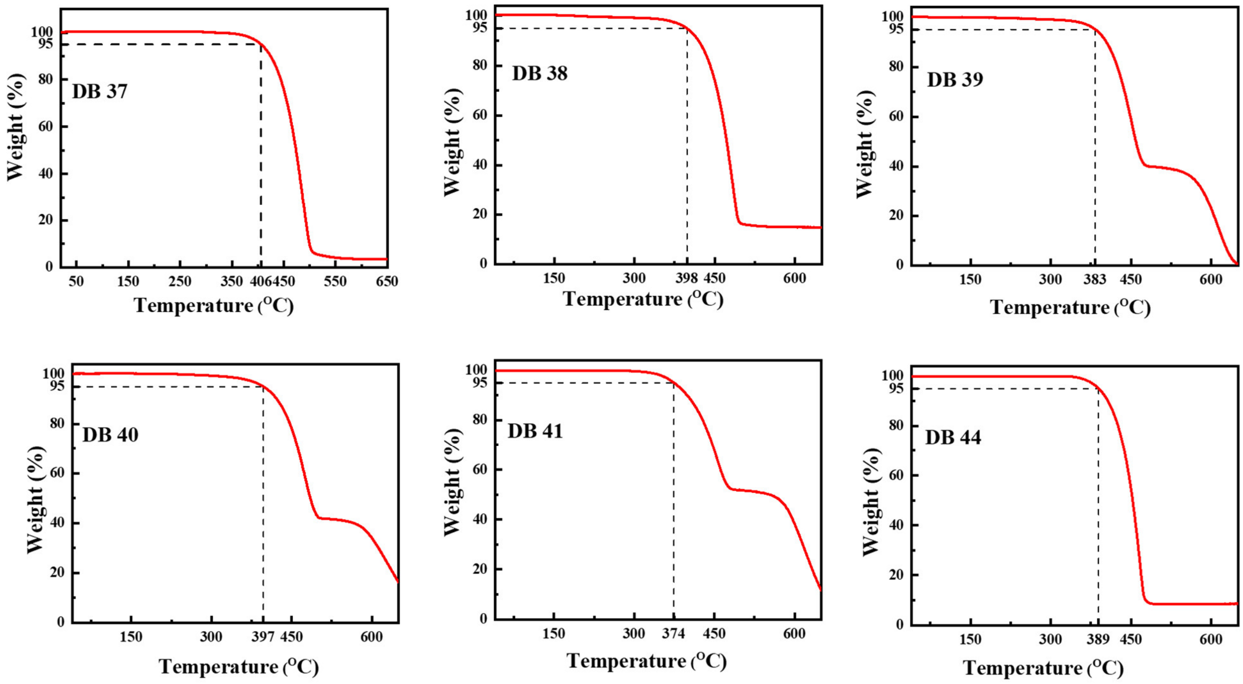

Figure S1. Data of thermogravimetric analysis of the compounds DB37 – DB41 and DB44. Heating rate: 10 °C/min.

2. UV-Vis absorption bands and Tauc plots

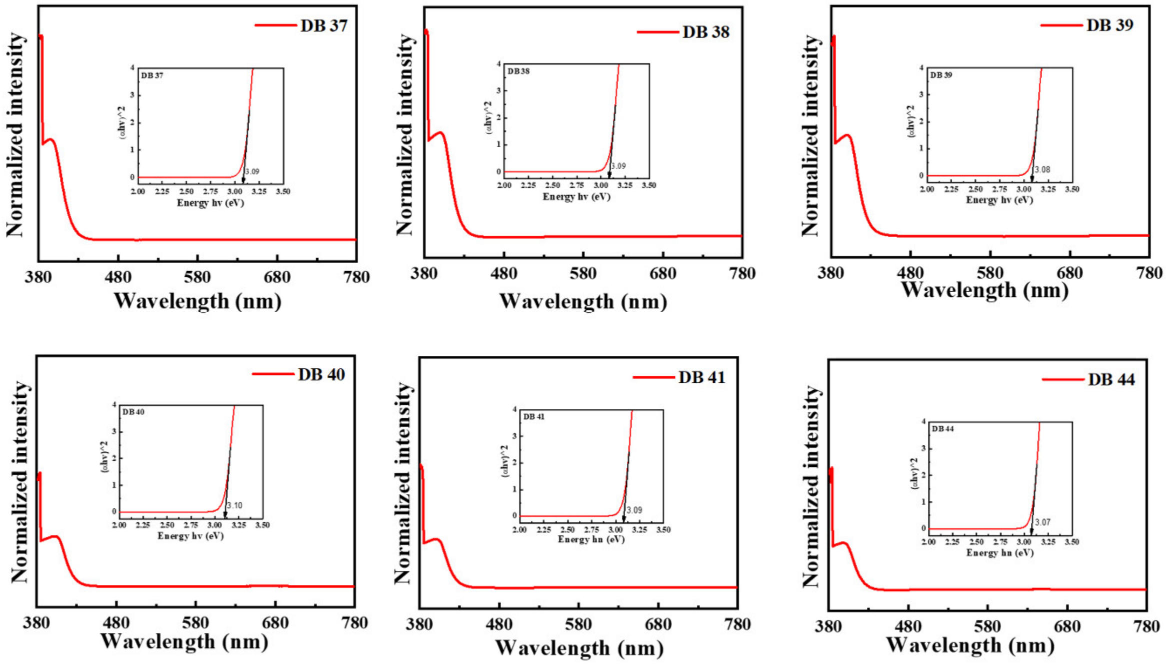

Figure S2. Ultraviolet-visible absorbance spectra and tauc plots (inset) of the compounds DB37 – DB41 and DB44.

3. Triplet energy

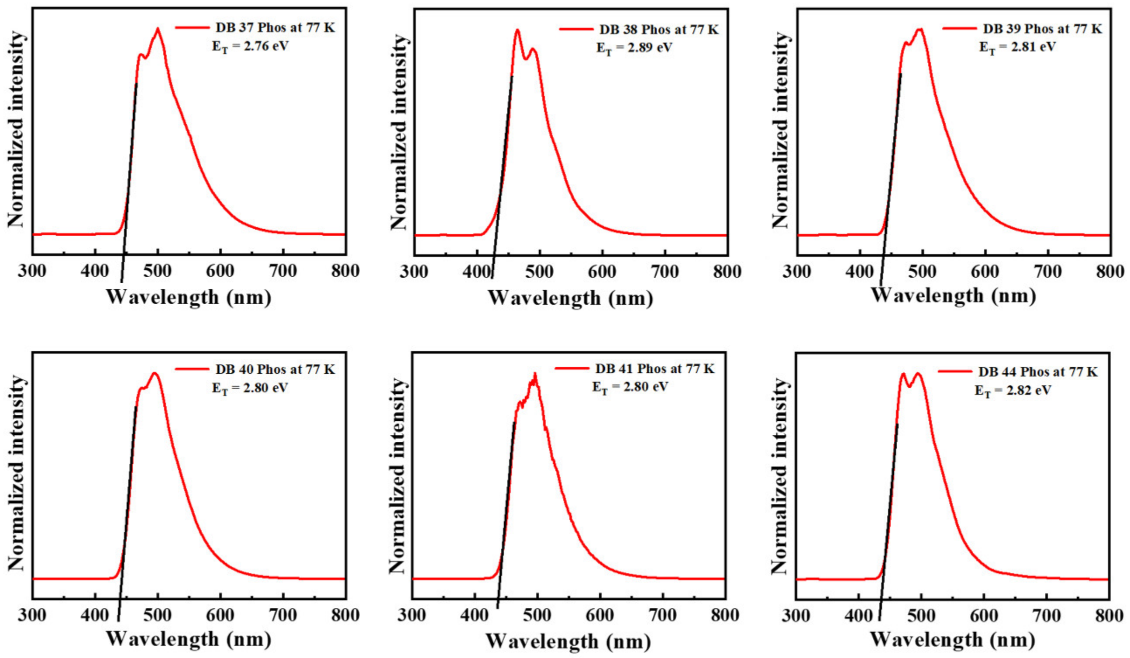

Figure S3. LTPL spectra of the derivatives DB37, DB38, DB39, DB40, DB41 and DB44 at 77K of the compounds.

4. Electroluminescent properties

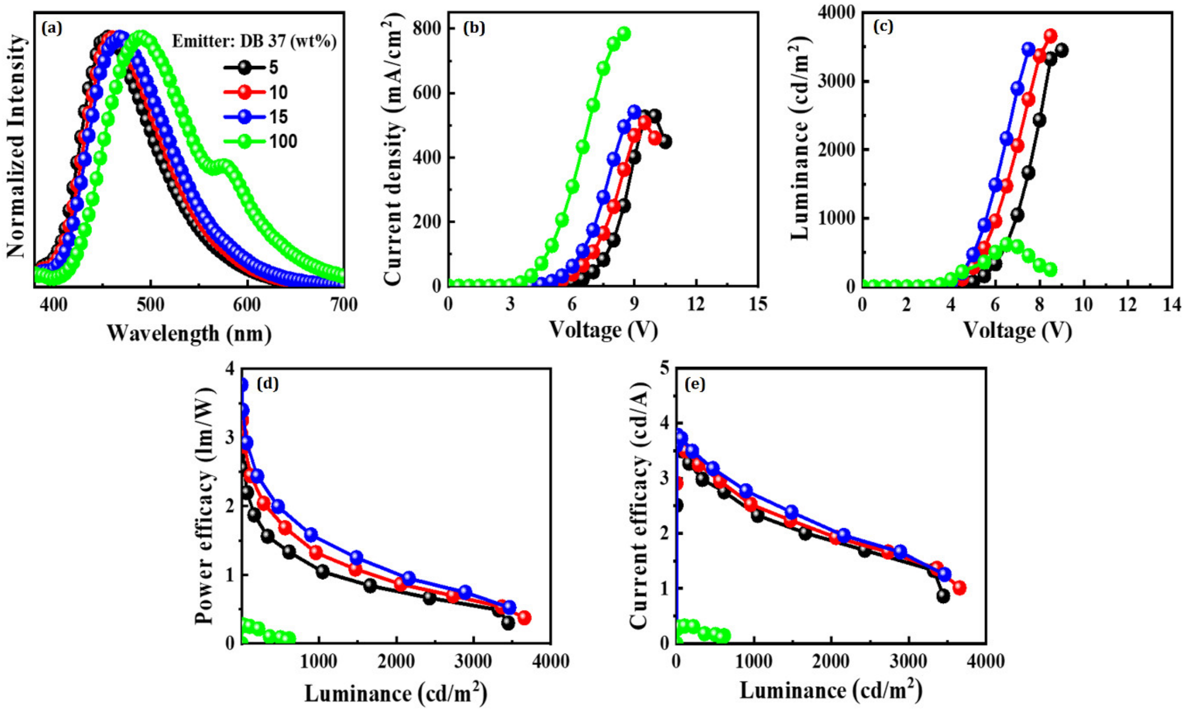

Figure S4. The electroluminescent (EL) properties of the device with emitter DB37 doped in CBP host matrix at varying concentrations showing (a) EL spectra, (b) current density–voltage, (c)

luminance–voltage, (d) power efficacy–luminance, and (e) current efficacy–luminance characteristics.

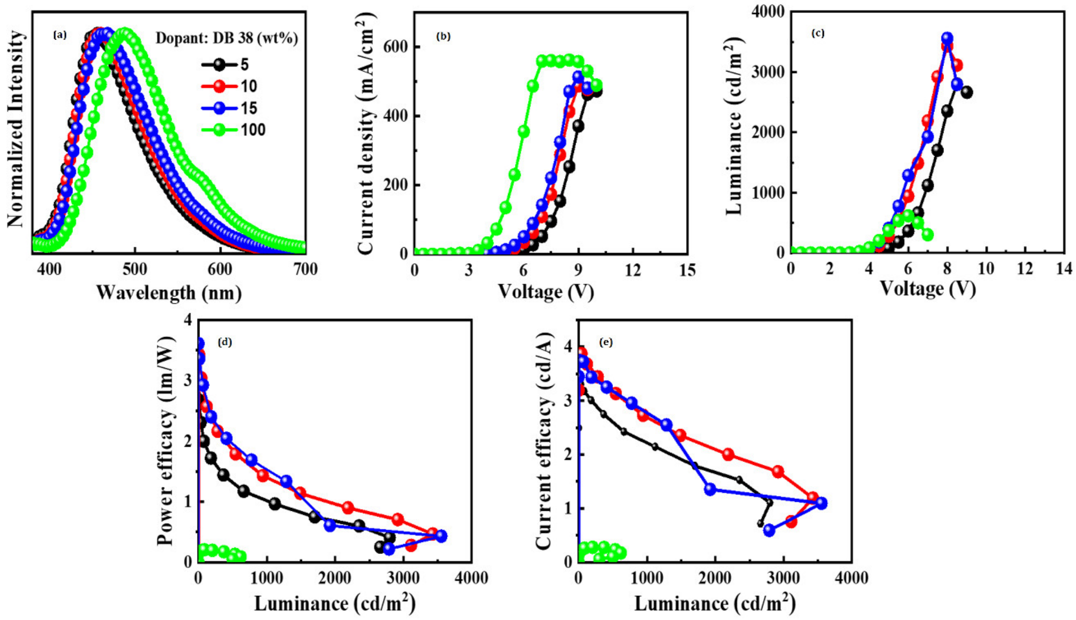

Figure S5. The electroluminescent (EL) properties of the device with emitter DB38 doped in CBP host matrix at varying concentrations showing (a) EL spectra, (b) current density–voltage, (c) luminance–voltage, (d) power efficacy–luminance, and (e) current efficacy–luminance characteristics.

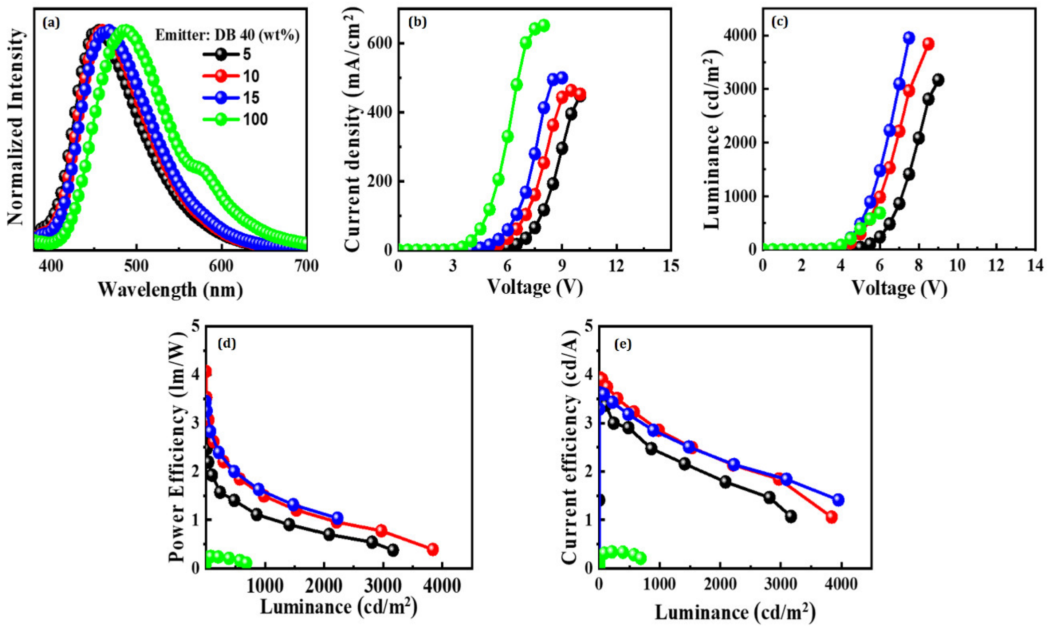

Figure S6. The electroluminescent (EL) properties of the device with emitter DB40 doped in CBP host matrix at varying concentrations showing (a) EL spectra, (b) current density–voltage, (c) luminance–voltage, (d) power efficacy–luminance, and (e) current efficacy–luminance characteristics.

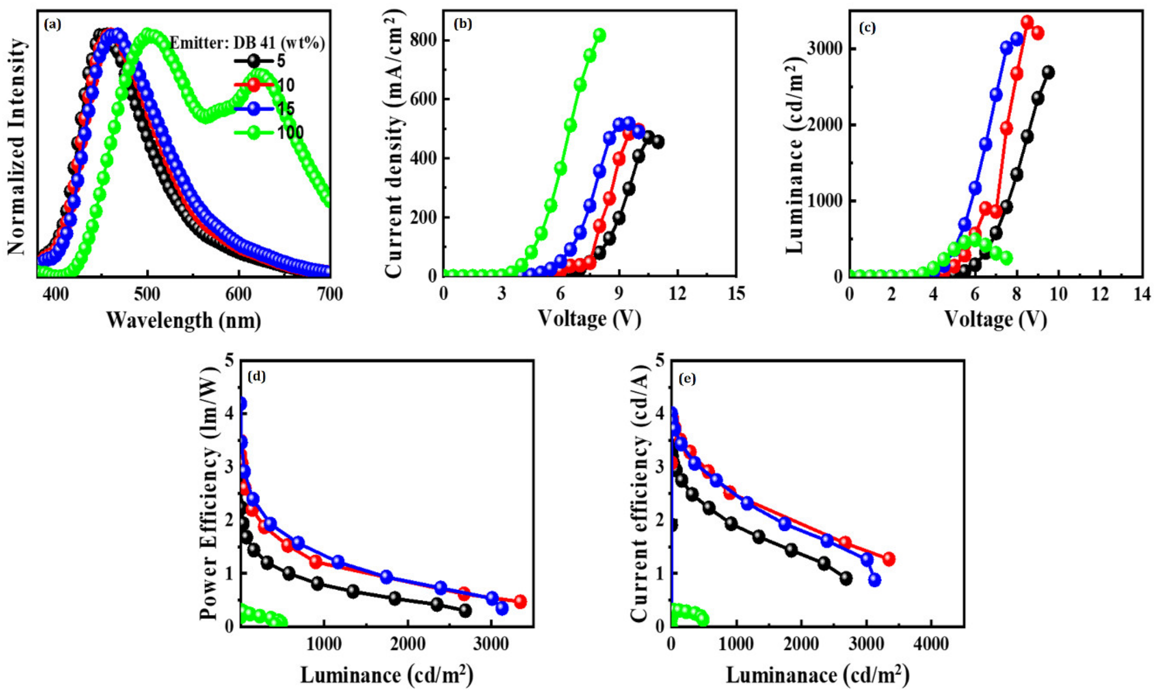

Figure S7. The electroluminescent (EL) properties of the device with emitter DB41 doped in CBP host matrix at varying concentrations showing (a) EL spectra, (b) current density–voltage, (c) luminance–voltage, (d) power efficacy–luminance, and (e) current efficacy–luminance characteristics.

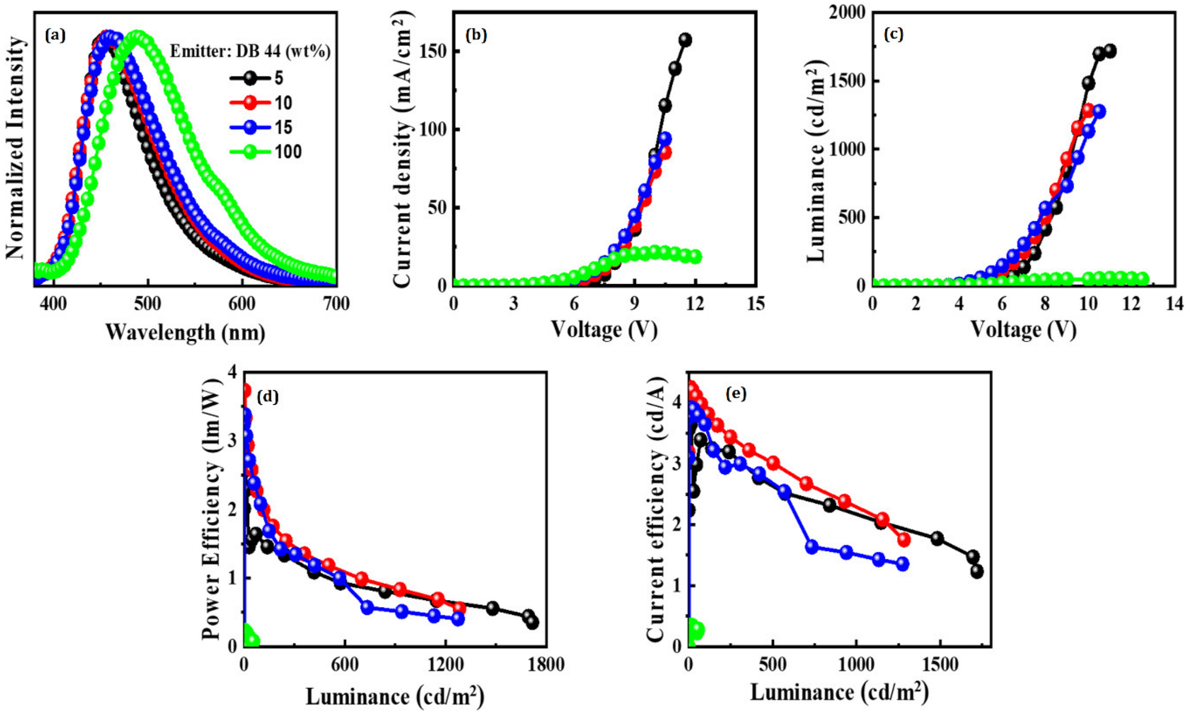

Figure S8. The electroluminescent (EL) properties of the device with emitter DB44 doped in CBP host matrix at varying concentrations showing (a) EL spectra, (b) current density–voltage, (c) luminance–voltage, (d) power efficacy–luminance, and (e) current efficacy–luminance characteristics.
